# 3D Flower-like *β*-MnO_2_/Reduced Graphene Oxide Nanocomposites for Catalytic Ozonation of Dichloroacetic Acid

**DOI:** 10.1038/srep43643

**Published:** 2017-03-02

**Authors:** Gang Li, Kezheng Li, Aijuan Liu, Ping Yang, Yukou Du, Mingshan Zhu

**Affiliations:** 1 College of Chemistry, Chemical Engineering and Materials Science, Suzhou 215123, P.R. China; 2 Medical College, Soochow University, Suzhou 215123, P.R. China; 3 The Institute of Scientific and Industrial Research (SANKEN), Osaka University, Osaka 567-0047, Japan

## Abstract

Considering the potential use of manganese oxide based nanocomposite in catalytic ozonation of water contaminant, we report unique three-dimensional (3D) nanoarchitectures composed of β-MnO_2_ and reduced graphene oxide (RGO) for catalytic ozonation of dichloroacetic acid (DCAA) from drinking water. The catalytic results show that the 3D β-MnO_2_/RGO nanocomposites (FMOG) can be used as efficient and stable ozonation catalysts to eliminate DCAA from water. The probable mechanism of catalytic ozonation was also proposed by detecting intermediates using gas chromatography-mass spectrometry. This result likely paves a facile avenue and initiates new opportunities for the exploration of heterogeneous catalysts for the removal of disinfection by-products from drinking water.

Recently, manganese oxide (MnO_x_) as an eco-friendly and inexpensively catalyst used in water treatment has caught more and more researchers’ attention^1^,^2^,^3^,^4^. Among various crystal phases of MnOx, β-MnO_2_, not only has the highest stability and the lowest water-solubility but also can be easily fabricated in various morphologies such as nanowires, nanorods, nanotubes and nanoflowers^5^,^6^,^7^,^8^,^9^,^10^. However, the catalytic performance of single-component β-MnO_2_ so far is unsatisfactory. In order to further enhance the catalytic property of β-MnO_2_, some promising methods such as modifying β-MnO_2_ with noble metals or combining β-MnO_2_ with other oxides have been developed^5^,^6^,^7^,^8^,^9^,^10^. For example, Wang *et al*. reported that gold nanoparticles modified β-MnO_2_ demonstrated enhanced catalytic activity for oxidizing alcohols to corresponding aldehydes or ketones^9^. Nevertheless, the high cost and limited resources of noble metals restricted the large scale application in the catalytic systems. Recently, graphene and its partial oxide counterpart-reduced graphene oxide (RGO) have emerged as promising candidates for fabricating new materials due to their high specific surface area, chemical stability, as well as biocompatibility^11^,^12^,^13^,^14^. It has been proved that composite fabricated by coupling graphene or RGO with semiconductors can achieve a higher catalytic activity.

Dichloroacetic acid (DCAA) as one of the disinfection by-products (DBPs) occurs in chlorinated drinking water systems^15^. United States Environmental Protection Agency (US EPA) has classified it as a possible carcinogenic compound^16^. In order to eliminate the DBPs including DCAA from drinking water sources, many advanced oxidation methods have been developed^17^,^18^,^19^,^20^. Among these methods, ozonation treatment of drinking water is regarded as one of the most promising techniques because it is cost-effective and there are no new DBPs to be produced. However, DCAA is hardly oxidized by ozone alone because the presence of the electron-withdrawing groups of -COO^−^ and -Cl in the molecule makes it extremely stable. An efficient ozonation catalyst should be developed to enhance the ozonation process.

Herein, we reported a unique three-dimensional (3D) flower-like nanocomposite (FMOG) consisted of pure β-MnO_2_ (PMO) and reduced graphene oxide (RGO). Interestingly, FMOG displayed higher catalytic performance compared with ozonation, and PMO catalytic ozonation. Considering the reaction conditions, such as pH, reaction temperature, and the dose of the catalyst, the FMOG nanocomposite was proved to be a robust and effective ozonation catalyst for eliminating trace DCAA from drinking water.

## Results

**Figure 1:**
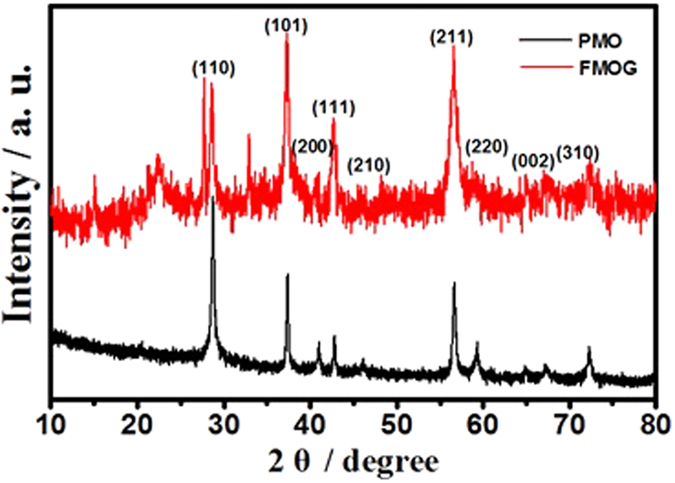
XRD patterns of PMO and FMOG.

Firstly, to validate the crystallinity of PMO and FMOG nanocomposites, the structure of our samples was investigated by X-ray diffraction (XRD), as shown in Fig. 1. For the sample of MnO_2_, the observed diffraction peaks locate at (110), (101), (200), (111), (210), (211), (220), (002), and (310) corresponding to reflections of the tetragonal β-MnO_2_ phase (JCPDS file NO. 24-0735) with the unit lattice a = b = 4.404 Å and c = 2.876 Å respectively^21^. For the FMOG nanocomposite, there was a small peak around located at around 22.5° besides the diffraction peaks of PMO, corresponding to the diffraction from (002) plane of graphene^22^. This result confirms the incorporation of reduced graphene oxide in the FMOG nanocomposites.

**Figure 2:**
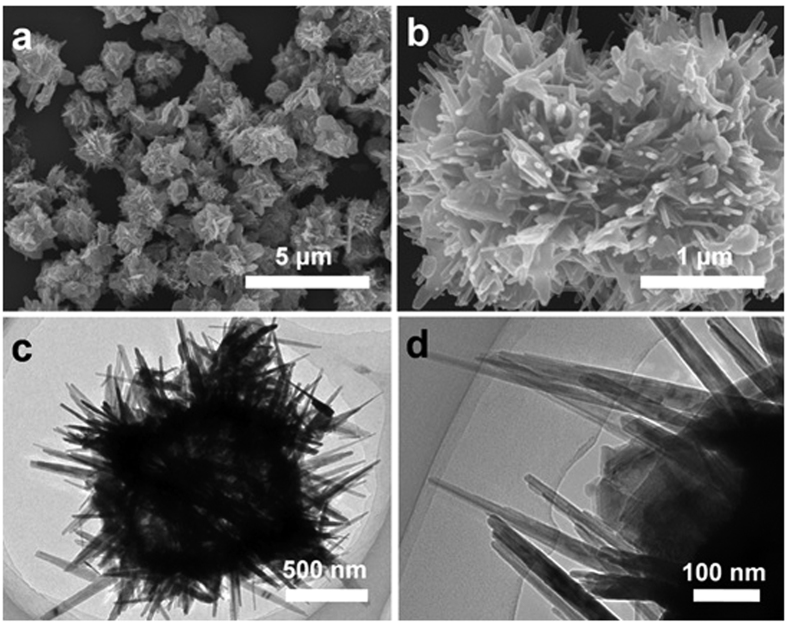
SEM (**a** and **b**) and TEM (**c** and **d**) images of FMOG nanostructures.

The morphology of prepared products was displayed by scanning electron microscopy (SEM) and transmission electron microscopy (TEM). The SEM and TEM of PMO are shown in Figure S1, the diameter of the PMO structures is around 1~2 μm and the diameter of single branch is about 20 nm. The SEM images reveal that the β-MnO_2_ crystal combined with RGO forming 3D flower-like β-MnO_2_/RGO structure, as shown in Fig. 2a and b. TEM images of FMOG further show 3D flower-like morphologies with multi-branch β-MnO_2_ and RGO nanosheets (Fig. 2c and d). The chemical component of FMOG was analyzed by energy dispersive X-ray (EDX) and X-ray Fluorescence (XRF) experiments, as shown in Figures S2 and S3. From EDX result, it can be seen that Mn, O and C elements are obvious detected in the samples and the semi-quantitative analysis shows that the atomic ratio between Mn and O element is ca. 1:2.1. This result indicates the formation of MnO_2_ in our samples. The atomic ratio between Mn and O is slight smaller than the theoretic stoichiometric ratio of MnO_2_, corresponding to O element in RGO. Besides Mn and O elements, carbon element can also be observed in the prepared sample, the mass ratio of C element in FMOG is ca. 14.5%, which suggests that the polylaminate reduced graphene oxide had been successfully coupled in the as-synthesized composites. Especially, the mass ratio of Mn element in FMOG is ca. 48.3% and 53% in XRF and EDX results respectively. This error may attribute to the difference of these two analysis methods.

To demonstrate the reduced graphene oxide in the nanocomposites, the Raman spectra of pure graphene oxide (GO) and FMOG are shown in Figure S4. The breathing mode of κ–point phonons of A_1g_ symmetry and E_2g_ phonons of sp^2^ C atoms in GO result in two obvious peaks of D- (1325 cm^−1^) and G-band (1598 cm^−1^) respectively^23^. After GO was hybridized with β-MnO_2_, the positions of D- and G-band shift to 1338 and 1591 cm^−1^, this might owe to the chemical environment change because of the introduction of β-MnO_2_ nanostructures in the composite. Moreover, the intensity ratio (I_D_/I_G_) in FMOG is about 1.1, which is obvious larger than that of the bare GO (*ca.* 0.9), suggesting the reduction of the sp^2^ network in RGO nanosheets^23^.

**Figure 3:**
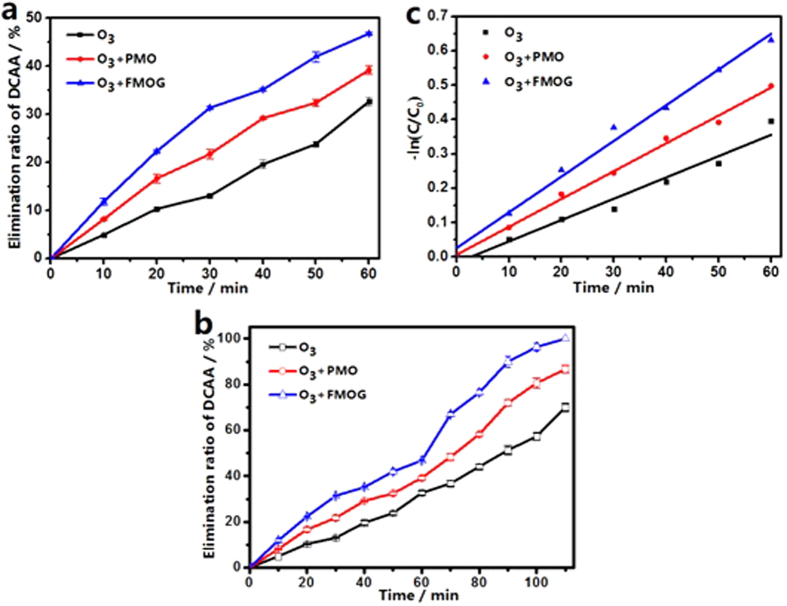
(**a**) Elimination of DCAA in solution by ozonation and catalytic ozonation for 60 minutes. (**b**) Elimination of DCAA in solution by ozonation and catalytic ozonation for 110 minutes. The flowing rate of O_3_ was 4.47 mmol min^−1^, the reaction temperature was 20 °C, the concentration of catalyst was 50 mgL^−1^, and the pH = 4.4. Error bars represent standard deviation of triplicate tests. (**c**) Logarithmic plot of the concentration of DCAA and linear fitting is present.

In order to eliminate the DCAA from drinking water sources, ozonation treatment of drinking water is regarded as one of the most promising techniques. The result of pure ozonation of DCAA showed (Fig. 3a) that 32.7% DCAA was degraded in 60 min. When the PMO or FMOG were added into the system, the elimination ratio of DCAA increased to 39.2% and 46.8% in 60 min respectively. Mehrjouei *et al*. reported that 90% DCAA has decomposed in 100 min by heterogeneous photocatalytic ozonation method^24^. In this research, as shown in Fig. 3b, DCAA was degraded completely in FMOG catalytic reaction after 110 min, while in the same reaction conditions the residual concentration ratio of DCAA was 30% and 13.3% for pure ozonation and PMO catalytic reaction. These results indicate an obvious catalytic ozonation effect of FMOG for DCAA degradation.

In all experiments, ozone was supplied into the reaction system superfluously and continuously. Therefore, the pseudo first order reaction (Equation (1)) is employed to investigate kinetics of the ozonation and the catalytic ozonation of DCAA:


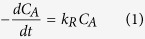


where *k*_*R*_ (min^−1^) is the apparent rate constant of the pseudo-heterogeneous catalytic ozonation. The logarithmic plot of the concentration of DCAA against reaction time is shown in Fig. 3c. For the pure DCAA ozonation process, the apparent rate constant is 0.0062 min^−1^. For the catalytic ozonation of DCAA process in the presence of PMO, the apparent rate constant augments to 0.0081 min^−1^. However, when using FMOG as catalyst, the apparent rate constant increases to 0.0104 min^−1^. Compared with *k*_*R*_ of the PMO catalytic process and the pure ozonation process, the apparent rate constant of FMOG catalytic process increases 28% and 68% respectively. The outstanding catalytic capacity of FMOG reveals that it can be used as an effective catalyst for the ozonation degradation of DCAA. Based on the previous researches^11^,^12^,^13^,^14^, RGO has π electron donating character of the *sp*^2^-bonded carbon structure, which can make the FMOG more effective than PMO in transferring electron to ozone^25^, and the polylaminate structure of RGO improved the surface area of the catalyst^26^, which provide more ozonation reaction centers. The synergistic effect of β-MnO_2_ and RGO in catalytic ozonation may contribute to the outstanding performance of FMOG.

Mass fragment ions of DCAA ozonation intermediates are detected by GC-MS simultaneously when the residual DCAA was analyzing and results are shown in Table 1. Product 1, 2 and 3 are possible molecular structures that were searched from NIST database. In the heterogeneous catalytic ozonation process, ozone reacts with OH^−^ to generate HO·^27^,^28^ via a chain reaction^29^,^30^ on the surface of catalyst (Equations 2 and 3), then HO· is regarded as the main oxidant of DCAA forming ·CCl_2_COO^−^ free radical and H_2_O, ·CCl_2_COO^−^ further reacts with oxygen forming peroxy radical ·OOCCl_2_COO^−^. The peroxy radical ·OOCCl_2_COO^−^ will decompose to carbonyl chloride which is unstable to water and decomposes to CO_2_ and HCl immediately (Equations (4, 5, 6, 7))^17^.


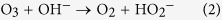



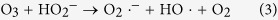



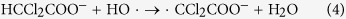



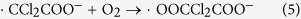



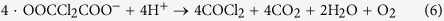



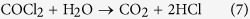


**Figure 4:**
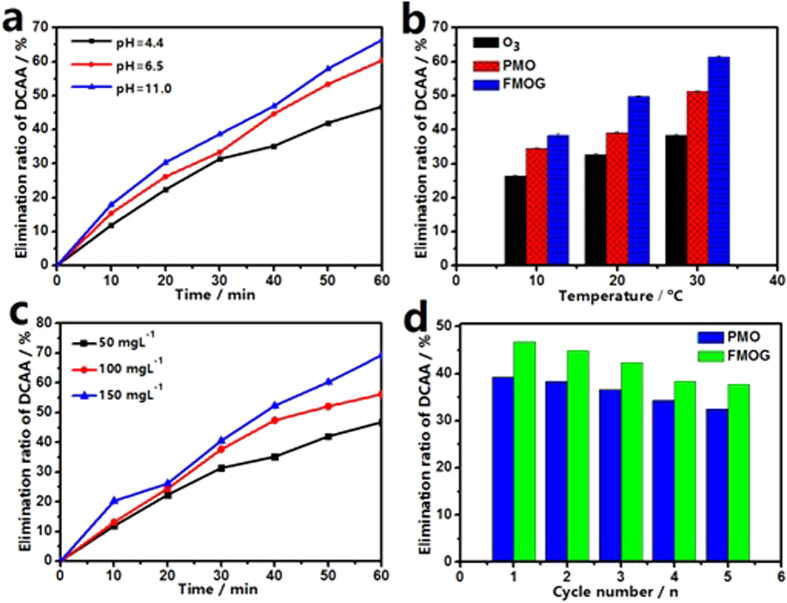
(**a**) Catalytic ozonation at different pH (4.4, 6.5, 11.0) using FMOG as a catalyst. (**b**) Ozonation and catalytic ozonation at different temperatures using PMO and FMOG as catalysts respectively. (**c**) Catalytic ozonation DCAA solution with FMOG at the different dose. (**d**) Elimination rate of recycling catalytic ozonation experiment using PMO and FMOG as catalysts. Catalytic ozonation condition: 200 mL DCAA solution (initial DCAA concentration was 100 mg L^−1^) in 60 min by using 50 mg L^−1^ (Fig. 4a,b and d) catalysts, pH = 4.4, the flowing rate of O_3_ was 4.47 mmol min^−1^, and the reaction temperature was 20 °C (Fig. 4a,c and d).

Usually, the pH of the reaction system plays a very important role in the catalytic ozonation process. Figure 4a shows the influence of initial pH of the solution to the catalytic ozonation DCAA by using FMOG as the catalyst. It can be seen that when the initial pH value of the system changes from 4.4 to 11.0, the elimination ratio of DCAA in 60 min increase from 46.8% to 66.4%, which can be attributed to the fact that the ozone decomposition rate is strongly pH dependent^31^. On the other hand, the pH point of zero charge (pH_PZC_) of the catalyst influences the adsorption of DCAA on the surface of FMOG (pH_PZC_ = 5.2) obviously. When the pH of the solution is below the value of pHpzc, the surface of the catalyst is positively charged, leading to electrostatic attraction between DCAA molecules and the catalyst since DCAA is a relatively strong acid and exists mainly in anionic form in aqueous solution^17^. This will bring adverse effect to the elimination of DCAA, because HO· radicals, which play a pivotal role in ozonation process, exist mainly in aqueous solution. As the pH of the solution increases, the number of the positive charge on the surface of the catalyst decreases, ultimately, the surface becomes negatively charged, this will reduce the adsorption of the substrate on the surface of the catalyst and enhance the degradation of DCAA.

On the other hand, the effect of the reaction temperature on the catalytic DCAA elimination is shown in Fig. 4b. As the reaction temperature augments from 10 °C to 30 °C, the elimination ratio of DCAA increases from 26.4% to 38.3% for pure ozonation. The same influence can be observed from the catalytic ozonation of DCAA, the elimination ratio of DCAA changes from 34.6% to 51.3% for PMO catalytic ozonation and 38.3 to 61.4% for FMOG catalytic reaction. It suggests that the increase of temperature accelerates the ozonation reaction, especially in catalytic ozonation.

The influence of the dose of the catalyst on the catalytic activity is shown in Fig. 4c. The DCAA elimination ratio at 60 min was improved from 46.8% to 69.3% when the dose of FMOG used in the system changed from 50 mg L^−1^ to 150 mg L^−1^, indicating that the catalytic activity of the catalysts promoted when the dose of catalyst increased. While, considering the situation of practical use, the right dose of the catalyst should be adopted in real applications.

Recycling experiments were carried out to estimate the stability of PMO and FMOG. The used catalyst was separated from the solution by centrifugation at 3500 rpm after 60 min of typical catalytic ozonation, and it was reused in the next catalytic ozonation after being washed by the distilled water and ethanol. As shown in Fig. 4d, the catalytic activity of FMOG decreases gradually in the first four runs. However, the catalytic activity of PMO decreases more obviously compared with the one of FMOG. In the fifth run, the DCAA elimination ratio of PMO dropped to 32.5%, demonstrating a continuous downtrend; while the DCAA elimination ratio of FMOG in the fifth run was 37.7%, which was almost the same as that of the fourth run. SEM of FMOG after fifth catalytic ozonation was shown in Figure S5, the flower-like nanostructure is still distinct. These facts indicate that FMOG is a relatively robust catalyst for catalytic ozonation of DCAA in practical applications.

In summary, 3D flower-like β-MnO_2_/RGO (FMOG) nanostructures have been successfully prepared and used as ozonation catalysts to eliminate DCAA from water. Compared to pure ozonation process, and β-MnO_2_ catalytic ozonation, the as-synthesized FMOG nanocomposite displayed highest catalytic performance. This is owing to the existence of RGO and the specific property of FMOG which was formed in synthesis process. The result might provide an approach to explore efficient and stable RGO composite for the catalytic ozonation of contaminant from drinking water sources.

## Methods

### Materials

All chemicals were purchased from China National Medicines Corporation and used as received without further purification. Ozone used for ozonation was generated in a laboratory ozone generator (Jingtian JT-XB-5P).

### Instruments

The morphologies of the catalysts were examined by a field scanning electron microscopy (SEM, Hitachi S-4800, Japan). The transmission electron microscopy (TEM) of the samples was investigated with a TECNAI G^2^ F-20 microscope operating at 200 kV. The energy dispersive X-ray (EDX) analysis was conducted with a Horiba EMAX X-act energy dispersive spectroscope that was attached to the S-4800 system. The X–ray diffraction (XRD) measurements were performed on a PANalytical X’ Pert PRO MRD system with Cu K_α_ radiation (λ = 1.54056 Å) operated at 40 kV and 30 mA. The X-ray Fluorescence (XRF) detections were performed on a Rigaku ZSX Primus II Spectrometer equipped with Al 25 optical filter. The Raman spectra were recorded on a Renishaw InviaPlus Raman microscope using a 633 nm argon ion laser. All of the measurements were carried out at room temperature.

### Synthesis

#### Synthesis of pure β-MnO_2_ (PMO) nanostructure

The pure β*-*MnO_2_ (PMO) was prepared through an ozonation and hydrothermal reaction. Ozone was bubbled into 25 mL of Mn(NO_3_)_2_ solution (50 g L^−1^) under magnetic stirring for 30 min. 20 mL of deionized water was added into the obtained dark suspension, and was stirred for another 30 min, then the suspension was transferred into a 50 mL autoclave with a Teflon-liner. The autoclave was heated to 200 °C and maintained at the temperature for 8 h. The powder sample was separated from the solution by centrifugation and washed with the ultrapure deionized water, and then dried in vacuum at room temperature.

#### Synthesis of three-dimensional (3D) flower-like β-MnO_2_/RGO (FMOG) nanocomposite

The nanocomposite was synthesized by three steps. Firstly, unilaminar GO nanosheet was synthesized by using chemical exfoliation of graphite with the modified Hummers’ method^32^. Then 0.5 mL of aqueous dispersion GO (1 mg L^−1^) was placed on a mold made of glass, and dried naturally in room temperature for 8 hours, this process repeated three times in the same mold, then the obtained polylaminate GO nanosheet (PGOS) can be peeled off from the mold after it dry thoroughly. Secondly a three-electrode system composed of an indium tin oxide (ITO) conductive glass covered PGOS as the working electrode, a platinum wire as the counter electrode and a saturated calomel electrode (SCE) as the reference electrode, and the electrodeposition of MnO_2_ on the PGOS as well as electrochemical reduction of PGOS were performed on a electrochemical workstation (CH Instrument Company CHI669D). The PGOS electrode was clamped by a conductive fixture and immersed in an aqueous solution of Na_2_SO_4_ (1.25 g L^−1^) and Mn(NO_3_)_2_ (0.75 g L^−1^), the potential range from 1.4 to −1.5 V at scan rate of 0.05 V s^−1^. The obtained MnO_2_/RGO substrate (MRS) was washed with deionized water and dried at room temperature, then it was further treated with a hydrothermal method to get 3D flower-like β-MnO_2_/RGO nanocomposite.

Thirdly 0.02 g of the as-obtained MRS was dispersed into 25 mL Mn(NO_3_)_2_ solution (50 g L^−1^), the mixture was treated by ultrasonic and ozone was bubbled for 30 min, then the suspension was transferred into a 50 mL autoclave with a Teflon-liner. The autoclave was heated to 200 °C and maintained at the temperature for 8 h. After the reaction, the powder sample was collected by centrifugal separation and washed thoroughly with ultrapure deionized water, and then dried in vacuum at room temperature, obtaining 3D flower-like β-MnO_2_/RGO (FMOG) nanostructures.

#### Ozonation and catalytic ozonation process

The typical catalytic ozonation of DCAA is similar to our previous work^33^. In details, the mixture of ozone and oxygen was bubbled into a 500 mL glass conical flask containing 200 mL of DCAA aqueous solution (100 mg L^−1^) with 10 mg of the as-prepared catalyst at room temperature under magnetic stirring. The flowing rate of O_3_ was 100 cm^3^ min^−1^ (4.47 mmol min^−1^). The initial pH of the catalytic system was adjusted by NaOH (0.05 mol L^−1^) or HCl (0.05 mol L^−1^). The initial concentration of DCAA was detected after adsorption equilibrium with the catalyst. In specified reaction intervals, 0.5 mL of DCAA solution was taken out from the conical flask during the reaction process. The taken solution was filtrated and then 0.1 mg of sodium thiosulfate and 0.1 mg of sodium sulphite were added to remove the residual O_3_. The concentration of residual DCAA and intermediates produced in ozonation were analyzed by a gas chromatography-mass spectrometry (Varian450GC-320MS) equipped with an electron ionization detector and HP-5 columns (0.25 μm, 30 × 0.25 mm) chosen selected ion monitoring (SIM) mode. Tentative identification of reaction products was achieved by comparing the authentic standards based on their retention times and mass spectra. The pH_PZC_ of FMOG was determined by the solid addition method.

## Additional Information

**How to cite this article**: Li, G. *et al*. 3D Flower-like *β*-MnO_2_/Reduced Graphene Oxide Nanocomposites for Catalytic Ozonation of Dichloroacetic Acid. *Sci. Rep.* **7**, 43643; doi: 10.1038/srep43643 (2017).

**Publisher's note:** Springer Nature remains neutral with regard to jurisdictional claims in published maps and institutional affiliations.

## Supplementary Material

Supplementary Information

## Figures and Tables

**Table 1:** Mass fragment ions (m/z) identified from GC–MS spectra,

	Detected ions (m/z)	Molecular structure
Product 1	127, 83	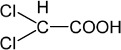
Product 2	84	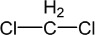
Product 3	98	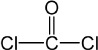
