# Supplementary material for: 3D Flower-like β-MnO2/Reduced Graphene Oxide Nanocomposites for Catalytic Ozonation of Dichloroacetic Acid
Source: Sci Rep. 2017 Mar 2;7:43643. doi: 10.1038/srep43643 (PMC5340795; doi:10.1038/srep43643)
Supplement: Supplementary Information [file srep43643-s1.docx]

**3D Flower-like *β*-MnO_2_/Reduced Graphene Oxide Nanocomposites for Catalytic Ozonation of Dichloroacetic Acid**

Gang Li,^†,‡^ Kezheng Li,^†^ Aijuan Liu,^†^ Ping Yang,^†^* Yukou Du,^†^ Mingshan Zhu,^§^*

^†^ College of Chemistry, Chemical Engineering and Materials Science, ^‡^ Medical College, Soochow University, Suzhou 215123, P.R. China

^§^ The Institute of Scientific and Industrial Research, Osaka University, Osaka 567-0047, Japan

* E-mail: pyang@suda.edu.cn (P.Y.); mingshanzhu@yahoo.com (M.Z.)


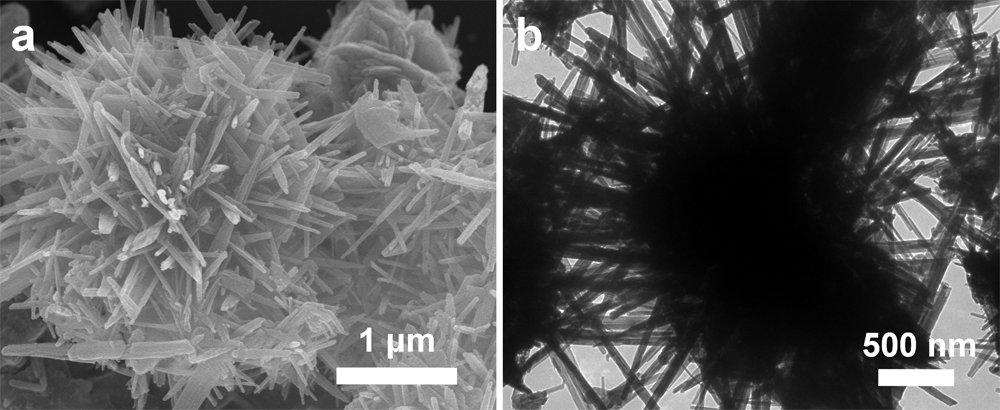


**Fig. S1.** SEM (a) and TEM (b) images of PMO nanostructures.


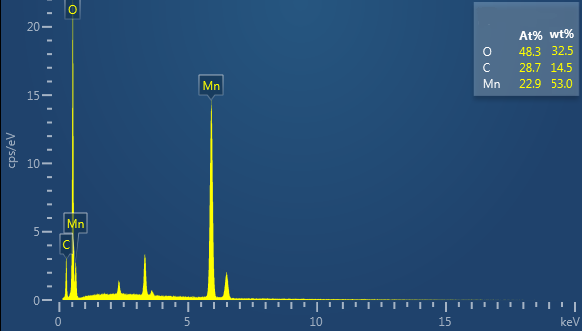


**Fig. S2.** EDX spectrum of FMOG.


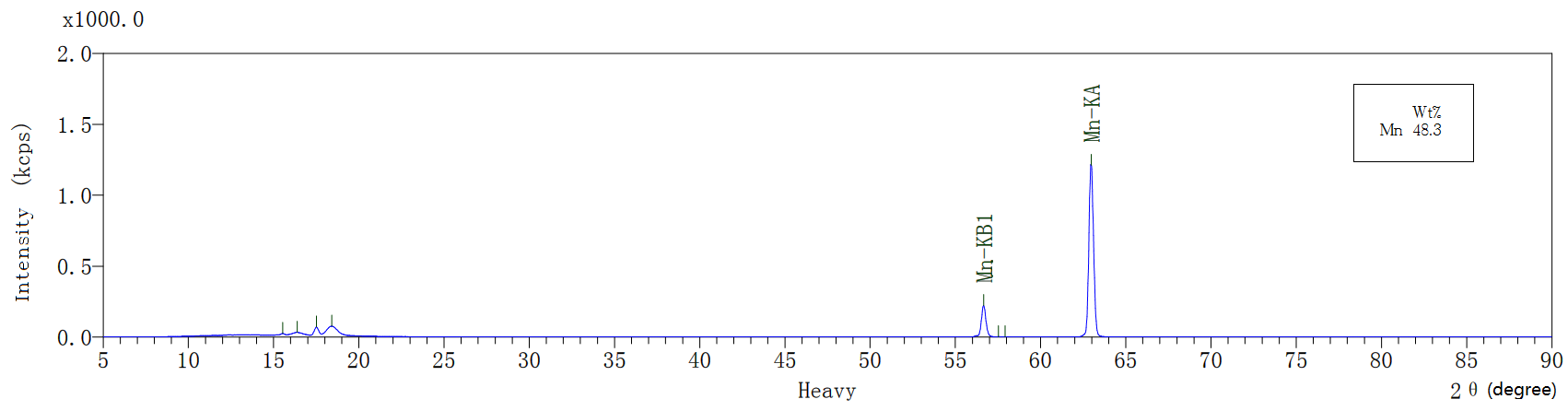


**Fig. S3.** XRF spectrum of FMOG.


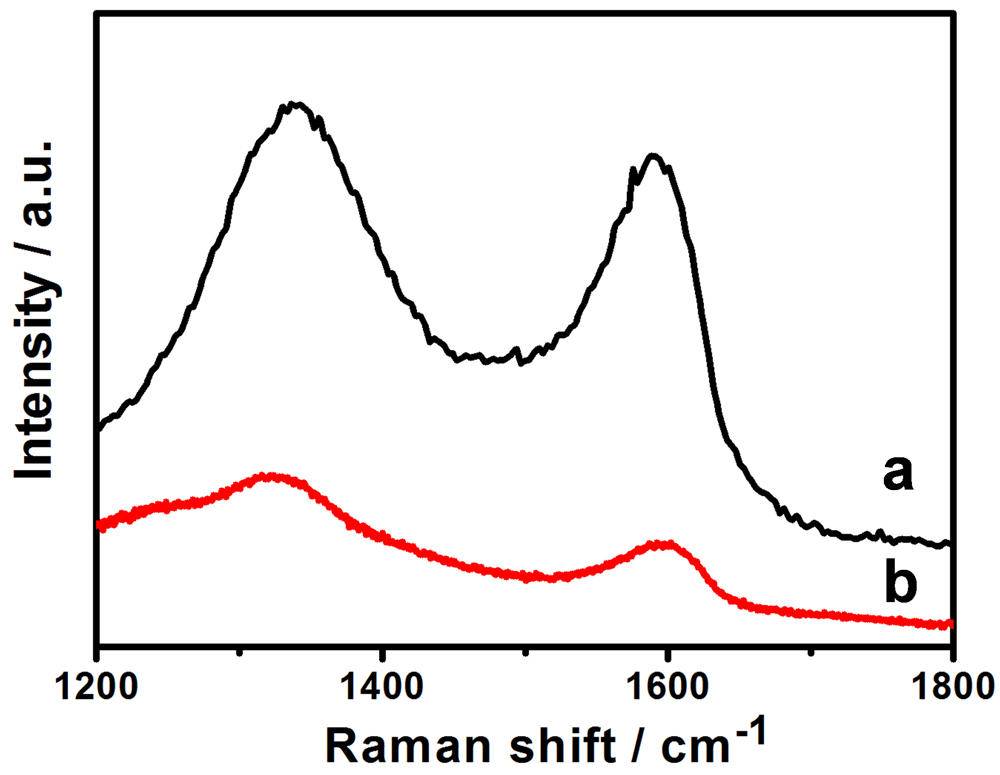


**Fig. S4.** Raman spectra of the pure GO sheets (a) and FMOG (b).


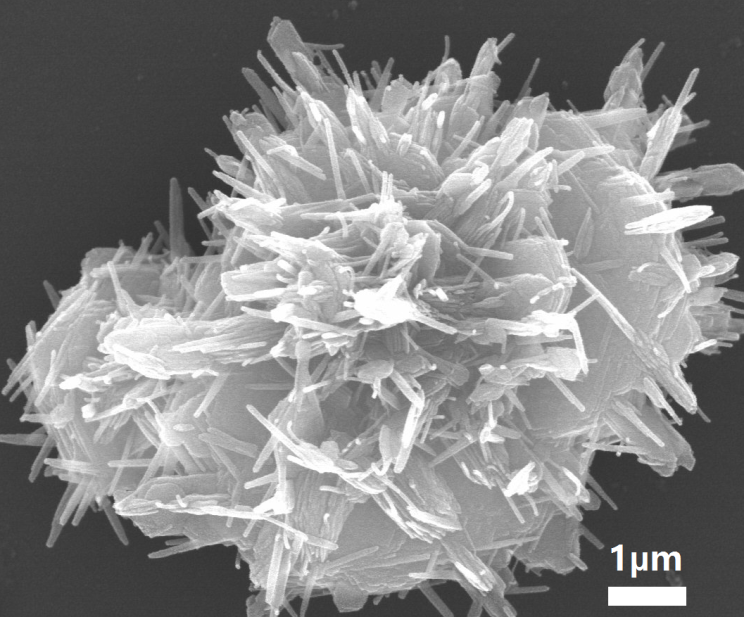


**Fig. S5.** EDX spectrum of FMOG after fifth catalytic ozonation.
